# Supplementary figures and images for: Probing the Limits to MicroRNA-Mediated Control of Gene Expression
Source: PLoS Comput Biol. 2016 Jan 26;12(1):e1004715. doi: 10.1371/journal.pcbi.1004715 (PMC4727922; doi:10.1371/journal.pcbi.1004715)

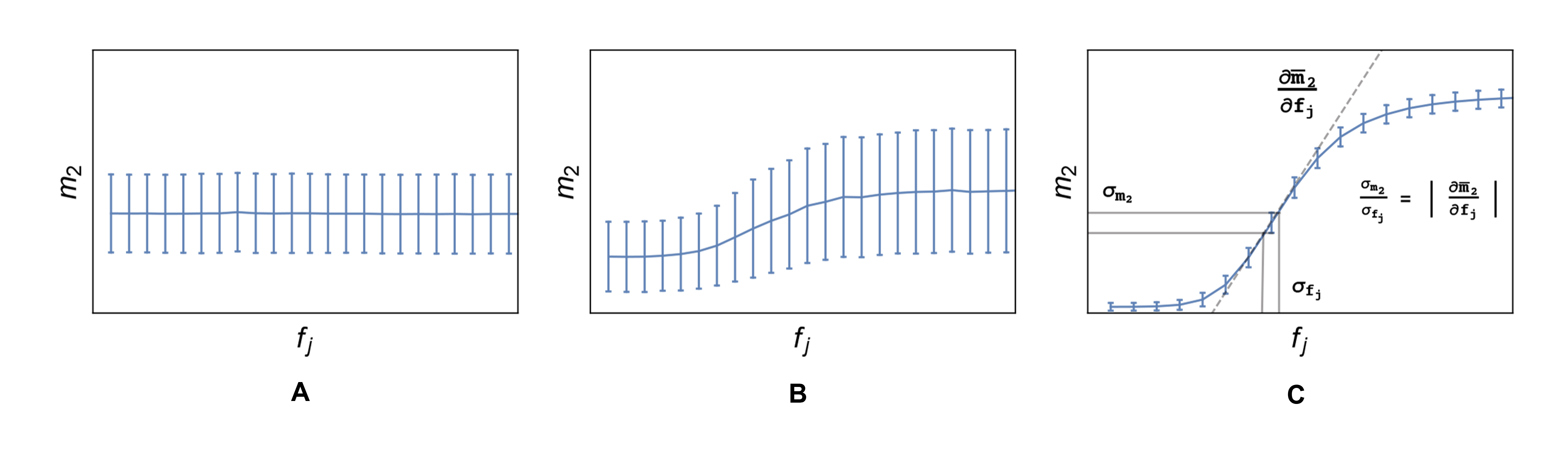

Supplement: S1 Fig — Qualitative depiction of how noise affects information flow. Three different situations for the input-output curve m 2 (target) vs f j (modulator) are shown, namely, long (A) target expression level independent of the modulator; (B) derepressed target, with large fluctuations; (C) derepressed target, with small fluctuations. In the first two cases, little or no information can be transmitted from modulator to target (hence the regulatory effectiveness is severely limited), as either the target is insensitive to changes in modulator levels, or its response is strongly hindered by noise. On the other hand, in case (C) information will be transmitted, since modulating the input one can clearly distinguish different output levels. The number of distinguishable levels is linked to the local slope of the input/output curve, as shown mathematically in Eqs (20) and (21) and is mainly limited by the noise strength. In the limit of vanishing noise, when the input-output relationship becomes deterministic, the mutual information between m 2 and f j diverges. (TIF) [file pcbi.1004715.s001.tif]

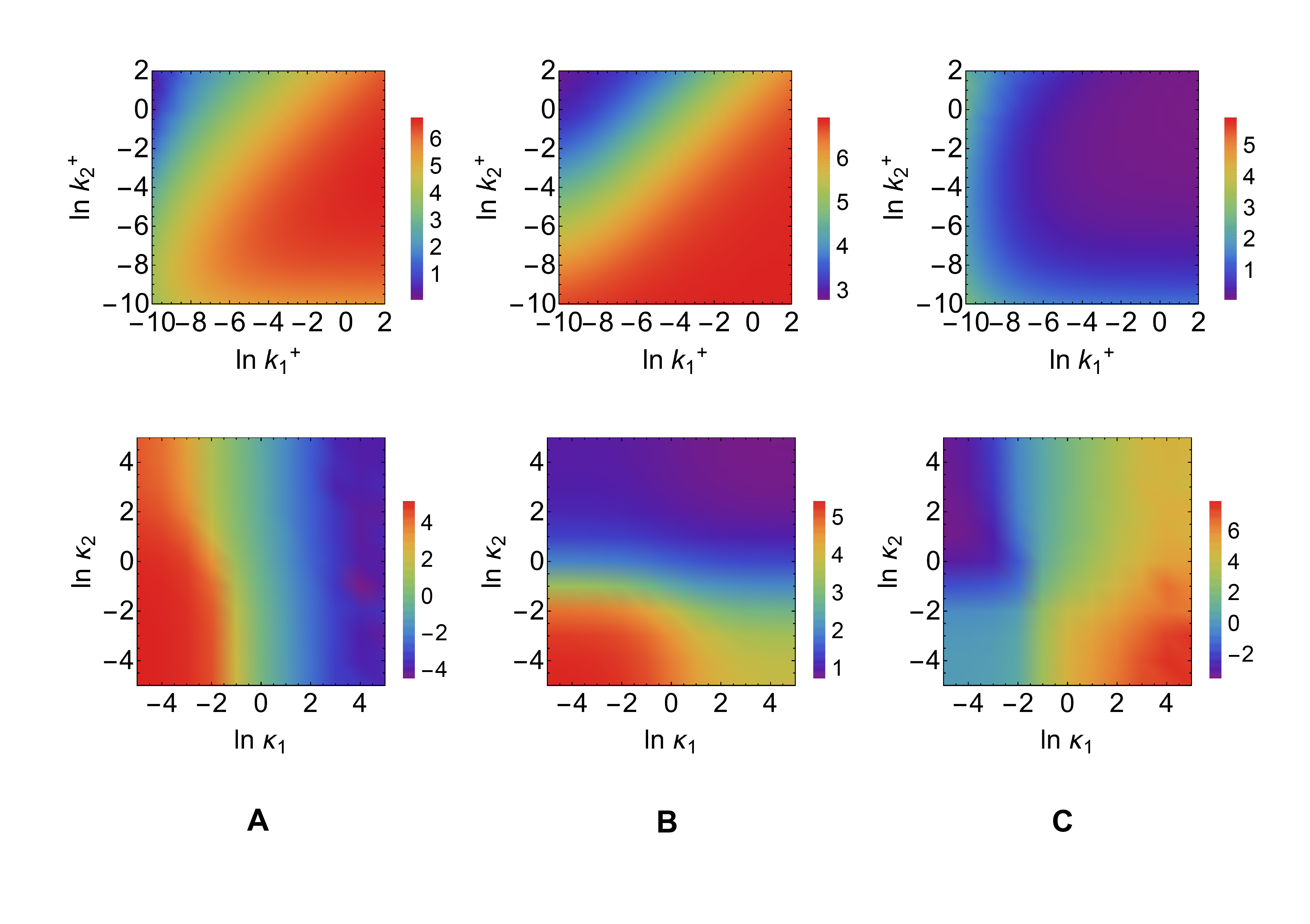

Supplement: S2 Fig — (A) ln ΔmiRNA, (B) ln ΔTF, (C) ln ΔTF—ln ΔmiRNA. Values of the kinetic parameters are as in Fig 5 for the panels in the top row and as in Fig 6 for the panels in the bottom row. (TIF) [file pcbi.1004715.s002.tif]

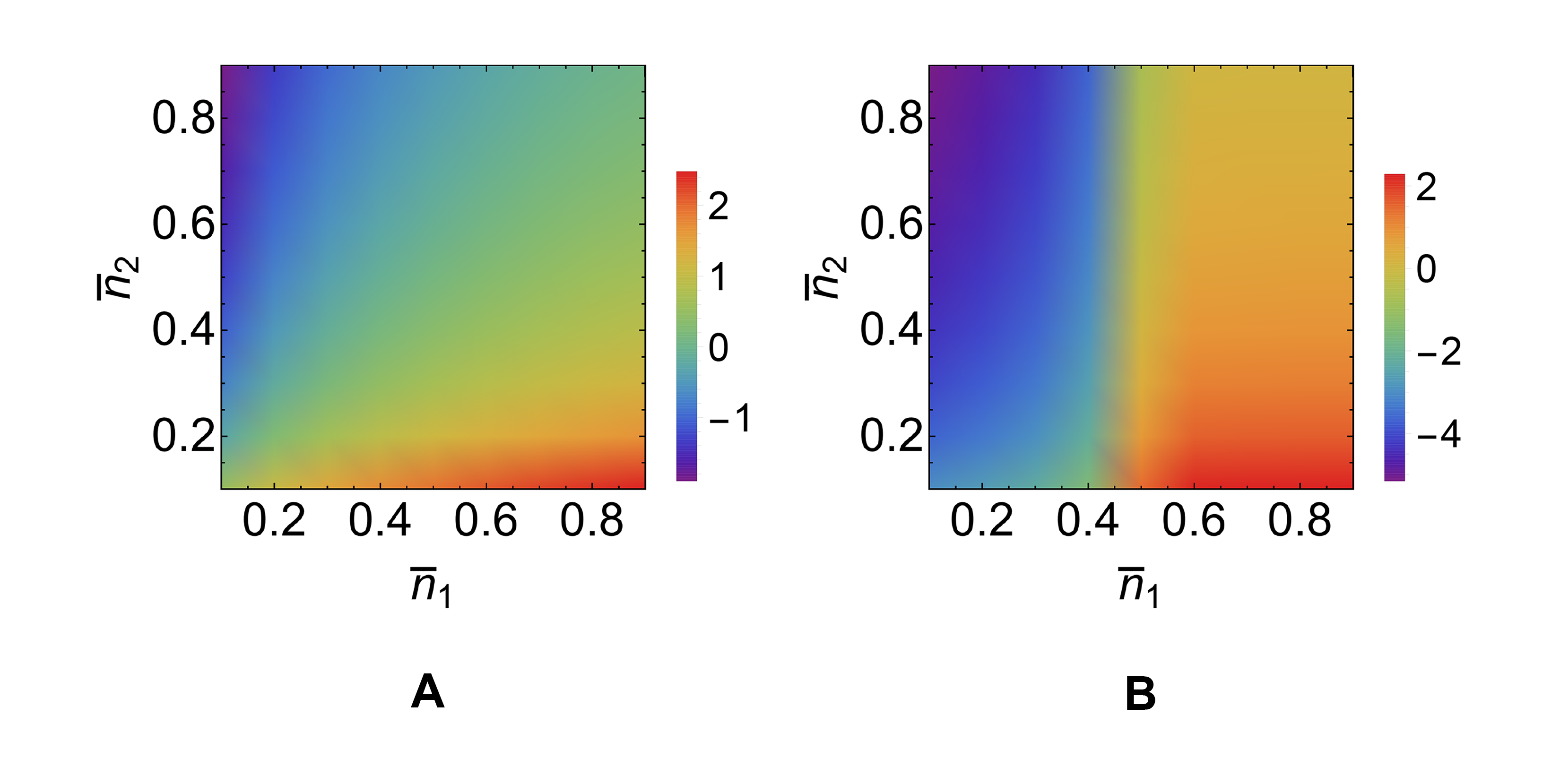

Supplement: S3 Fig — Values of the kinetic parameters are as in Fig 7A for panel (A), and as in Fig 7B for panel (B). (TIF) [file pcbi.1004715.s003.tif]
