# Supplementary material for: Probing the Limits to MicroRNA-Mediated Control of Gene Expression
Source: PLoS Comput Biol. 2016 Jan 26;12(1):e1004715. doi: 10.1371/journal.pcbi.1004715 (PMC4727922; doi:10.1371/journal.pcbi.1004715)
Supplement: S1 Text — (PDF) [file pcbi.1004715.s004.pdf]

S1 Text. Capacity of a Poissonian channel. In the absence of miRNA molecules, fluctuations in TF-channel are Poissonian (i.e. the corresponding FF is one). In such a case, the channel capacity (see Eq. 21) is given by

$$I_{\text{opt}} = \log_2 \frac{1}{\sqrt{2\pi e}} \int_{f_j^{\min}}^{f_j^{\max}} \frac{1}{\sqrt{\overline{m}_2}} \frac{d\overline{m}_2}{df_j} df_j = \log_2 \frac{1}{\sqrt{2\pi e}} \int_{m_2^{\min}}^{m_2^{\max}} \frac{d\overline{m}_2}{\sqrt{\overline{m}_2}} =$$
$$= \log_2 \left(\sqrt{m_2^{\max}} - \sqrt{m_2^{\min}} \right) - \frac{1}{2} \log_2 \frac{\pi e}{2} \quad ,$$

where $m_2^{\min} = \overline{m}_2(f_j^{\min})$ and $m_2^{\max} = \overline{m}_2(f_j^{\max})$ are the minimum and maximum expression levels of the target.
